# HBM4EU Chromates Study—Genotoxicity and Oxidative Stress Biomarkers in Workers Exposed to Hexavalent Chromium

**DOI:** 10.3390/toxics10080483

**Published:** 2022-08-18

**Authors:** Ana Tavares, Kukka Aimonen, Sophie Ndaw, Aleksandra Fučić, Julia Catalán, Radu Corneliu Duca, Lode Godderis, Bruno C. Gomes, Beata Janasik, Carina Ladeira, Henriqueta Louro, Sónia Namorado, An Van Nieuwenhuyse, Hannu Norppa, Paul T. J. Scheepers, Célia Ventura, Jelle Verdonck, Susana Viegas, Wojciech Wasowicz, Tiina Santonen, Maria João Silva, on behalf of the HBM4EU Chromates Study Team

**Affiliations:** 1 Department of Human Genetics, National Institute of Health Dr. Ricardo Jorge (INSA), Av. Padre Cruz, 1649-016 Lisbon, Portugal; 2 Finnish Institute of Occupational Health, 00250 Helsinki, Finland; 3 French National Research and Safety Institute, 54500 Vandœuvre-lès-Nancy, France; 4 Institute for Medical Research and Occupational Health, Ksaverska Cesta 2, HR-10001 Zagreb, Croatia; 5 Department of Anatomy Embryology and Genetics, University of Zaragoza, 50013 Zaragoza, Spain; 6 Centre for Environment and Health, Department of Public Health and Primary Care, KU Leuven (University of Leuven), O&N 5b, Herestraat 49, P.O. Box 952, 3000 Leuven, Belgium; 7 Department of Health Protection, Laboratoire National de Santé (LNS), 3555 Dudelange, Luxembourg; 8 IDEWE, External Service for Prevention and Protection at Work, 3001 Heverlee, Belgium; 9 Centre for Toxicogenomics and Human Health (Toxomics), NOVA Medical School, Universidade NOVA de Lisboa, Campo dos Mártires da Pátria, 130, 1169-056 Lisbon, Portugal; 10 Department of Environmental and Biological Monitoring, Nofer Institute of Occupational Medicine, 91348 Lodz, Poland; 11 HTRC—Health & Technology Research Center, ESTeSL—Escola Superior de Tecnologia da Saúde, Instituto Politécnico de Lisboa, 1549-020 Lisbon, Portugal; 12 Radboud Institute for Health Sciences, Radboudumc, 6500 HB Nijmegen, The Netherlands; 13 NOVA National School of Public Health, Universidade NOVA de Lisboa, 1600-560 Lisbon, Portugal; 14 Comprehensive Health Research Center (CHRC), 1169-056 Lisbon, Portugal

**Keywords:** effect biomarkers, micronuclei, comet assay, MDA, 8-OHdG, chromate, occupational exposure, biomonitoring

## Abstract

A study was conducted within the European Human Biomonitoring Initiative (HBM4EU) to characterize occupational exposure to Cr(VI). Herein we present the results of biomarkers of genotoxicity and oxidative stress, including micronucleus analysis in lymphocytes and reticulocytes, the comet assay in whole blood, and malondialdehyde and 8-oxo-2′-deoxyguanosine in urine. Workers from several Cr(VI)-related industrial activities and controls from industrial (within company) and non-industrial (outwith company) environments were included. The significantly increased genotoxicity (*p* = 0.03 for MN in lymphocytes and reticulocytes; *p* < 0.001 for comet assay data) and oxidative stress levels (*p* = 0.007 and *p* < 0.001 for MDA and 8-OHdG levels in pre-shift urine samples, respectively) that were detected in the exposed workers over the outwith company controls suggest that Cr(VI) exposure might still represent a health risk, particularly, for chrome painters and electrolytic bath platers, despite the low Cr exposure. The within-company controls displayed DNA and chromosomal damage levels that were comparable to those of the exposed group, highlighting the relevance of considering all industry workers as potentially exposed. The use of effect biomarkers proved their capacity to detect the early biological effects from low Cr(VI) exposure, and to contribute to identifying subgroups that are at higher risk. Overall, this study reinforces the need for further re-evaluation of the occupational exposure limit and better application of protection measures. However, it also raised some additional questions and unexplained inconsistencies that need follow-up studies to be clarified.

## 1. Introduction

Multiple industrial applications of Cr(VI) provide benefits to our society but have also resulted in adverse side effects in humans and the environment [1]. Occupational settings in which exposure to Cr(VI) occurs include welding with Cr-containing metals and alloys, electroplating, and other surface treatment processes (e.g., both painting and removal of old Cr-containing paint), are the most important chromium-related industrial activities [2,3]. The inhalation of dusts, mists, or fumes from Cr-containing products is the main route of occupational exposure [4] and the respiratory tract is the main target of Cr(VI)-related adverse health effects [1,5,6]. Dermal contact and ingestion due to hand-to-mouth contact are routes of exposure that are also considered relevant within the occupational context [7,8] and may contribute to effects that are elicited at the skin, immune, and reproductive systems [9,10]. There is strong evidence associating lung and other respiratory tract cancers with occupational exposure to Cr(VI) [11,12,13,14]. Those evidences led to the inclusion of Cr(VI) compounds in the international Agency for Research on Cancer (IARC) Group 1, i.e., carcinogenic to humans [3].

Cr(VI)-induced carcinogenesis involves the deposition and accumulation of Cr(VI) particles in the bifurcations of the airways, when the capacity of clearance and detoxification mechanisms is exceeded [13] being distributed to nearly all tissues [15]. Most of the Cr(VI) is reduced to Cr(III) by glutathione and ascorbate and accumulates mainly in the kidney, liver, and bone tissues of humans and rodents [4], increasing the body’s internal load. Cr(VI) has been shown to exert toxic and genotoxic effects, both in vitro and in vivo [3], and to be mutagenic [16]. If absorbed into the cell, a reduction of Cr(VI) to Cr(III) generates intermediate Cr species that induce DNA and protein changes and reactive oxygen species (ROS) that may lead to oxidative DNA damage. In addition, both pathways can result in DNA single and double strand breaks, and chromosomal alterations [1,3,17]. ROS accumulation contributes additionally to chronic inflammation, metabolic reprogramming, and genetic instability, ultimately leading to tumor development [18,19,20]. Epigenetic alterations, decreases in DNA repair signaling, telomere alterations, and aneuploidy that are related to Cr exposure may also contribute to cell transformation [3,21,22,23,24,25]. As proposed by Nickens et al. (2010), the selection of cells with the ability to survive after exposure to apoptogenic levels of Cr(VI) may yield a precursor pool of cells harboring altered DNA repair and survival signaling mechanisms from which neoplastic variants may emerge, leading to tumor formation [26].

Despite the recognized health effects and the strict regulatory measures to manage exposure to Cr(VI) at workplaces, including the need for authorization (a temporary permit) for the continued use of hexavalent chromium compounds in the EU (Annex XIV of REACH regulation), Cr(VI) is still much used in the metal sector in the EU. This is the consequence of the lack of appropriate substitutes for some of its industrial applications. In the EU, a binding occupational exposure limit value (BOELV) for the ambient air concentration of Cr(VI) of 0.005 mg/m^3^ has been given (Directive 2017/2398). However, a transitional period was defined until 2025 during which the limit value of 0.010 mg/m^3^ is applied. For welding fumes, a limit value of 0.025 mg/m^3^ is in place during the same transitional period, after which the generally applicable limit value of 0.005 mg/m^3^ will become the binding limit. Nationally, France has derived the most stringent OEL of 1 μg/m^3^ [27] and corresponding biological limit value of 2.5 µg/L for chromium plating workers. The same OEL is applied in the Netherlands [28] and in Denmark [29].

Concentrations of Cr in blood and urine have long been used for biological monitoring of environmentally- or occupationally-exposed populations [30]. Whereas urinary Cr (U-Cr) is nonspecific for Cr(VI) and reflects both past and recent exposure, the measurement of Cr in red blood cells (RBC-Cr) has been considered to assess intracellular Cr(VI) exposure occurring during the lifespan of the RBC; Cr measurement in plasma (P-Cr) reflects the systemic level of Cr(III) [31,32]. The inclusion of effect biomarkers is also important to characterize, at an early stage, the potential impact of Cr(VI) on workers’ health [5,31]. It has been suggested that Cr(VI) exposure may result in different health effects depending on whether it is used as an end-product (chromate production), as a start-product (electroplating), or as a by-product (welding) [33], which might also relate to different levels of exposure. Also, from this perspective, effect biomarkers can be useful to differentiate the bioactivity of Cr(VI) according to its use. A recent review by Ventura et al. (2021) [5] concluded that the most often used effect biomarkers in Cr(VI)-related occupational studies have targeted oxidative stress and genotoxicity, particularly those measuring DNA and chromosomal damage, such as the comet and the micronucleus assays in blood leukocytes, respectively.

The micronucleus (MN) assessment in cytokinesis blocked (CBMN) human peripheral blood lymphocytes (MN PBL), comprises of the analysis of several parameters indicating structural and/or numerical chromosome alterations or cytostasis (MN cytome assay) and has been used as a predictor of cancer risk for over 20 years [30,34]. Flow cytometric analysis of MN in peripheral blood reticulocytes (MN RET) is one of the most recent tools in human biomonitoring. It is a sensitive high-throughput method with high potential in biomonitoring studies [35]. The method has been previously used for studying the potential genotoxicity of pesticides [36], disinfection by-products [37], industrial pollution [38], and polycyclic aromatic hydrocarbons (PAHs) [39]. As application of flow cytometry allows for the rapid analysis of large amounts of cells, the method also has benefits compared to microscopically analyzed endpoints which suffer from the limited number of reasonably countable cells and potential individual differences in analyzing cells [40]. The Comet Assay or the Single Cell Gel Electrophoresis (SCGE), is a rapid, simple, and sensitive technique for measuring and analyzing recent DNA breakage in individual cells [41]. It has found numerous applications in occupational biomonitoring studies [42,43]. Oxidative stress is a complex and incompletely understood process that involves a variety of cellular changes. Therefore, no single biomarker can serve as a complete measure of this complex biological process. Amongst the oxidative stress biomarkers that were analyzed in Cr-exposed workers, urinary malondialdehyde (MDA) levels or oxidative DNA adducts, such as 8-hydroxydeoxyguanosine (8-OHdG) were the most frequently reported [5], as opposed to blood antioxidant enzymes, for example. Similar to other effect biomarkers, also these oxidative stress markers are not specific for Cr exposure and thus the potential influence of other exogenous or endogenous factors cannot be excluded.

Some occupational studies have included genotoxicity biomarkers and/or oxidative stress biomarkers, mainly displaying positive results. A significantly higher frequency of MN PBL [1,44,45] and MN in exfoliated buccal cells of chrome plating workers [46], has been reported. Similarly, there are studies suggesting an association between welders’ Cr(VI) exposure and induction of DNA damage and its repair inhibition [47,48,49]. A study that was focused on chromate production workers also showed significantly higher MN PBL frequency, and urinary 8-OHdG levels that were associated with elevated air and blood Cr levels [50].

Under the European Human Biomonitoring Initiative (HBM4EU, www.hbm4eu.eu (accessed on 17 June 2022)), a multi-center cross-sectional study involving workers from several activities with potential exposure to chromates was performed, aiming to collect new data on current occupational exposure to Cr(VI). The chromates study offered the unique opportunity to include the assessment of a set of effect biomarkers in a study comprising of an unusually large number of participants, covering different industrial sectors and activities, and with a carefully planned design to provide harmonized and comparable data on external and internal Cr(VI) exposure, besides the studied early biological effects. The present study aimed to characterize a set of well-established effect biomarkers in a subset of workers that were enrolled in the aforementioned study, comprising the analysis of MN PBL, MN RET, the comet assay in leukocytes, and oxidative stress biomarkers (MDA and 8-OHdG) in urine. The potential relationships between exposure and effect biomarkers as well as among the different effect biomarkers that were studied, were also explored.

## 2. Materials and Methods

### 2.1. Workplace and Study Population

In the planning phase of this multicenter study, standard operating procedures (SOPs) were prepared to ensure the harmonization of recruitment, materials and procedures for collection, handling, storage, and transfer of the biological samples [32]. Companies from nine countries, having in common workers with potential exposure to Cr(VI) were included. Such activities encompassed stainless-steel welding, bath chrome plating, surface treatment by sanding, spraying or painting using chromate-containing paints, thermal spraying using heated metallic Cr (with possible formation of Cr(VI) fumes), steel production and machining, maintenance and laboratory work in chrome plating, or surface treatment companies. As controls, workers from the same industries but without predictable exposure to Cr(VI), e.g., administrative staff (within company controls), were selected. In addition, workers from other companies with no activities that were associated with Cr(VI) exposure (outwith company controls) were recruited in two countries (Portugal and Finland) to complete the subset of control individuals. Details about companies that were involved, work distribution, and the samples that were collected by country for environmental and internal exposure monitoring have been previous published [31,32]. Ethics approval was secured from each participating country’s ethics committee. Each company and potential participant were informed about the procedures and the objectives of the study, and those who agreed to participate provided written informed consent for the collection and utilization of biological specimens. All the study participants answered a questionnaire gathering sociodemographic data, as well as data on lifestyle, occupational, and medical history. More detailed information about the overall study design, including a scheme of the workflow, can be found in Santonen et al. [31].

Blood samples for effect marker analyses were collected in five out of nine participating countries (Belgium, Finland, Netherlands, Poland, and Portugal). Urine samples for oxidative stress biomarkers assessment (Table 1) were collected in Belgium, Finland, Netherlands, Poland, and France. Healthy adult subjects (18–70 years) from both genders, who were present at work during the study period, were included. Smokers and ex-smokers (for at least 6 months before sampling) were included in both groups, despite the knowledge that tobacco smoke is one of the major sources of Cr(VI) environmental exposure. Participants who were or had been on treatment for cancer (n = 9) and those who had been submitted to X-ray examinations or Computerized Axial Tomography Scans in the last three months (n = 25) were excluded, as previously defined in the standard operating procedures [31].

Workers from several industrial sectors performing an array of activities that were associated with different levels of Cr(VI) exposure were included. To account for those differences, the exposed and control groups that were targeted in this study were further categorized in subgroups, according to the same general criteria that were previously described [32]. As such, the following subgroups of workers were considered: bath plating workers, chromate paint applicators, welders, and machining workers; to encompass workers that were involved in thermal spraying, steel production and maintenance and laboratory tasks (in chrome plating or surface treatment), another subgroup, designated as “other activities”, was created, due to the low number of individuals that were involved in each of these activities. As each country workers’ recruitment was dependent on the types of companies and workers willingness to participate, variations in task distribution among countries were noted. For example, in some countries, welders were predominantly involved whereas in others bath platers or chromate paint applicators were mainly recruited. The control group included both “within company controls”, and “outwith company controls”, i.e., controls that were selected within the same industry or outside the industry, as detailed in the previous section.

### 2.2. Biological Samples Collection and Biomarkers Analyses

Blood sample collections were preferentially performed on the 3rd–5th day of the working week. Briefly, for effect biomarker measurements, a minimum of 6 mL of peripheral blood was collected by venipuncture, distributed in 2 tubes with sodium-heparin anticoagulant and kept at 4–10 °C, and protected from light during storage and transportation. Blood collection, processing, and measurement of exposure biomarkers in plasma and red blood cells was performed according to the procedures that were described by Ndaw et al. [51].

There were two spot urine samples that were collected from the exposed workers, the first one before the start of the shift or at the beginning of the working week, and the second one at the end of the shift or at the end of the working week. One spot urine sample was collected from the control individuals at any time of the working week. Given that urine samples were also used for Cr measurement, they were collected in previously decontaminated containers (pre-washed with 10% of nitric acid solution) to avoid background contamination. After collection, the samples were homogenized and aliquoted in several pre-labelled tubes and stored at −20 °C before analysis.

U-Cr analysis was performed by laboratories that had successfully passed a Quality Assurance/Quality Control (QA/QC) Program comprising several rounds of interlaboratory comparison investigations (ICI) [52,53]. Either inductively coupled plasma-mass spectrometry (ICP-MS) or graphite furnace atomic absorption spectrometry (GFAAS) were used. The urinary creatinine concentrations were also measured, and the Cr results were normalized to creatinine.

The effect biomarker analysis was centralized in three laboratories, each one receiving, processing, and providing the data for all the samples that were collected for a given biomarker, to prevent interlaboratory variations. As such, blood samples for analysis of MN PBL and for comet assay in blood leukocytes were handled by a single laboratory (INSA, PT), blood samples for MN RET analysis were handled by another one (FIOH, FI), and a third laboratory (INRS, FR) processed the urine samples for MDA and 8-OHdG measurements.

### 2.3. Cytokinesis-Blocked Micronucleus (CBMN) Assay in Lymphocytes

The MN assay in human PBL was performed as previously described [54]. Briefly, 2 whole blood cultures were set up from each donor in RPMI-1640 medium that was supplemented with fetal bovine serum (15%) and phytohemagglutinin A (PHA, 2.5%) (all from Thermo Fisher Scientific, Waltham, MA, USA), for 68 h at 37 °C. At 44 h, cytochalasin B (Sigma-Aldrich, St. Louis, MO, USA) at 5 µg/mL was added to each culture flask to block cytokinesis. At 68 h, the lymphocytes were harvested by treatment with a pre-warmed hypotonic solution (KCl 0.1 M) (Merck, Darmstadt, Germany), followed by two fixation steps with methanol: acetic acid (Merck, Darmstadt, Germany) solutions (3:1 and 97:3). The cells were immediately dropped onto microscope slides and after air-drying they were stained with a 4% Giemsa solution (Merck, Darmstadt, Germany) in pH 6.8 phosphate buffer. For each blood sample, at least two replicate cultures were performed. In addition, at least 2 extra cultures from randomly selected blood samples were treated with mitomycin C (MMC) (Sigma-Aldrich, St. Louis, MO, USA) at 43 h at a final concentration of 0.01 μg/μL and incubated for 1 h before cytochalasin B addition, to work as positive controls. MN were blindly scored, under a bright field microscope with a 400–1000× magnification, and identified according to published criteria [55]. For each individual, at least 2000 cytokinesis-blocked lymphocytes (appearing as binucleated cells, BC) with well-preserved cytoplasm were scored in slides from the 2 cultures for MN, nucleoplasmic bridges (NPBs), and nuclear buds (NBUDs), according to the published criteria [55,56]. The results of micronucleated binucleated cells (MNBC), MN in BC, NPBs, and NBUDs were expressed as frequencies per 1000 BC. The proportion of mono- (MC), BC, or multi-nucleate cells (MTC) was determined in a total of 1000 cells per participant and the cytokinesis-block proliferation index (CBPI) was calculated as follows [57]: CBPI = (MC + 2BC + 3MTC)/Total cells.

### 2.4. Micronucleus (MN) Assay in Reticulocytes (RET)

The MN assay in transferrin-positive peripheral blood RET was performed according to the method that was developed by Abramsson-Zetterberg et al. [35] as previously described [39]. Briefly, the whole blood samples were processed within 7 days after collection and transferrin-positive (+CD71) RET were isolated by immunomagnetic separation according to the instructions of the CELLection™ Pan Mouse IgG Kit (Invitrogen, Thermo Fisher Scientific, Waltham, MA, USA), using a FITC Mouse Anti-human CD71 antibody (BD Biosciences, San Jose, CA, USA). Thereafter the samples were fixed in 2% paraformaldehyde in PBS with 10 µg/mL of sodium dodecyl sulfate (SDS; Sigma-Aldrich, Merck KGaA, Darmstadt, Germany) and kept refrigerated (4 °C) until analysis. Prior to the analysis, DNA was stained with Hoechst 33342 (Invitrogen, Thermo Fisher Scientific, Walthamt, MA, USA). The samples were analyzed using blue (488 nm) laser for the identification of +CD71 RET and near UV (375 nm) laser for the detection of DNA-containing MN. A CytoFlex S flow cytometer and CytExpert software version 2.3 (Beckman Coulter, Brea, CA, USA) were used for data acquisition and analysis. The MN frequency (MN RET) was quantified as per-mille of micronucleated +CD71 RET from all analyzed +CD71 RET. A minimum of 20,000 +CD71 RET per sample were required to ensure reliable data, resulting in the exclusion of seven samples.

### 2.5. Comet Assay

The alkaline version of the comet assay was used to evaluate DNA damage in peripheral blood leukocytes from the participants and was carried out as previously described [54]. Briefly, 20 µL of blood cells were embedded in 0.7% low melting point agarose (Sigma-Aldrich, St. Louis, MO, USA) and placed onto microscope slides that were previously coated with 1% normal melting point agarose (GE Healthcare Life Sciences, Sweden). A positive control was included in all assays consisting of blood cells exposed to ethyl methanesulfonate (EMS) (Sigma-Aldrich, St. Louis, MO, USA) at 30 mM for 30 min at 37 °C. The microscope slides were then immersed in a fresh cold lysis solution [89% NaCl (2.5 M), Na_2_EDTA.2H_2_O (100 mM), Tris-HCl (10 mM), NaOH (10 M); 1% Triton X-100; 10% DMSO] (all from Merck, Darmstad, Germany) and incubated for 1–14 h at 4 °C. After lysis, the slides were covered by electrophoresis buffer [NaOH (300 mM); Na_2_EDTA.2H_2_O (1 mM); pH > 13] and allowed to unwind for 20 min. Electrophoresis was then performed for 20 min at 4 °C, with amperage and voltage defined at 300 mA and 25 V, respectively. After the electrophoresis, the slides were washed three times with neutralization buffer [Trizma-base (Sigma-Aldrich, St. Louis, MO, USA) 0.4 M in deionized water, with 9.5% vol HCl (Merck, Darmstad, Germany) 4 M; pH = 7.5] for 5 min at 4 °C. The slides were kept in a box, protected from the light, to dry at room temperature until analysis. The slides were stained with ethidium bromide (125 μg/mL) (Sigma-Aldrich, St. Louis, MO, USA), and observed under a fluorescence microscope (DM2500, Leica Microsystems CMS GmbH, Mannheim, Germany), with the assistance of specific image-analysis software (Comet Assay IV, Perceptive Instruments, Cambrige, UK). A total of 100 nucleoids were randomly analyzed per sample (50 nucleoids per slide, 2 gels per sample). The amount of DNA damage was expressed by the percentage of the DNA in the tail (or tail intensity) of the nucleoids given the existence of a linear relationship between both parameters [58]. Other parameters, such as tail length and tail moment were also determined.

### 2.6. Oxidative Stress Biomarkers

The protocol that was used for the determination of 8-OHdG was adapted from the method that was described and published by Hosozumi et al. [59]. Briefly, a urine sample (2 mL) was applied on solid phase extraction cartridge (Oasis MCX 3cc 60 mg, Waters) that was previously conditioned with methanol and water. The cartridge was washed with water and the analytes were eluted with 3 mL of methanol then evaporated to dryness standard (under a stream of nitrogen). The residue was redissolved in 1 mL water: acetonitrile (1:9, *v*/*v*), and an aliquot of 5 µL was injected into the LC-MS/MS system. The samples were analyzed on a Shimadzu 8030 liquid chromatography triple quadripole mass spectrometer. Chromatographic separation was achieved on a Sequant^®^ZIC^®^-HILIC column (100 × 2.1 mm, 5 µm) from Merck with a mixture of 5 mM ammonium acetate/ACN 1% (solvent A) and acetonitrile (solvent B) as a mobile phase. The MS/MS system operated in positive mode. Signal acquisition was performed in SRM mode monitoring with the ion transitions: 282.2–192.1 m/z for 8-OHdG and 287.20–197.1 *m*/*z* for [15N]8-OHdG as an internal standard. The limit of quantification (LOQ), determined as the lowest concentration, which can be quantified with accuracy within 20% of the nominal value and a precision which should not exceed 20%, was 0.2 µg/L.

The protocol that was used for the determination of MDA was adapted from the method that was described and published by Chen et al. (2011) [60]. Briefly, a urine sample (100 µL) was derivatized with 895 µL of 0.5 mM of DNPH and 5 µL of a solution of 1 mg/L of d2-MDA at 50 °C for 5 h. The sample was then diluted 10 times with water and an aliquot of 20 µL was injected into the LC-MS/MS. The samples were analyzed on a Shimadzu 8030 liquid chromatography triple quadripole mass spectrometer. Chromatographic separation was achieved on a Kinetex C18 column (100 × 2.1 mm, 2.6 µm) from Phenomenex with a mixture of water (solvent A) and acetonitrile (solvent B) as mobile phase. The MS/MS system operated in positive mode. Signal acquisition was performed in SRM mode monitoring with the ion transitions: 235.0–159.05 *m*/*z* for DNPH-MDA and 237.0–161.1 *m*/*z* for DNPF-d2-MDA. The LOQ, determined as the lowest concentration which can be quantified with an accuracy within 20% of the nominal value and a precision which should not exceed 20%, was 1 µg/L.

### 2.7. Statistical Analysis

The results of the effect biomarkers (MN PBL, MN RET, comet assay, and oxidative stress biomarkers) were expressed by the mean and standard deviation (SD). The normality of the distribution of the effect biomarkers values among exposure groups was assessed using the Shapiro–Wilk and the Kolmogorov–Smirnov tests, while the homogeneity of variance was evaluated by the Levene test. The differences between the exposure groups were analyzed using the non-parametric Kruskal–Wallis (KW) and Mann–Whitney (MW) U tests, since all effect biomarkers that were assessed did not present a normal distribution. Pre-shift and post-shift MDA and 8-OHdG levels were assessed using the signed-rank Wilcoxon test. Non-parametric (MW or KW) tests were applied to explore the influence of several socio-demographic and lifestyle factors (e.g., country, age, gender, tobacco or alcohol consumption, residence in industrial or urban areas, etc.) on the dependent variables that were evaluated, i.e., MN PBL, MN RET, and comet tail intensity. The impact of other exposure determinants, such as the companies scale or the work history in chromium-related jobs was not evaluated due to the fact that the information on these variables was incomplete for a great proportion of the participants.

Multiple linear regression analyses were subsequently applied to confirm the influence of independent variables on the effect biomarker levels and to assess differences between the exposed and control groups, after adjusting for the effect of country, gender, and alcohol consumption that showed the strongest influence.

The correlations between the different effect biomarkers or between effect and exposure biomarkers were investigated using Spearman correlations.

Statistical analyses were performed using IBM SPSS Statistics for Windows, Version 26.0 (IBM Corp. Released 2019, Armonk, NY, USA). A *p*-value that was lower than 0.05 was considered statistically significant.

## 3. Results

### 3.1. Characteristics of the Study Population

Due to collection refusal, technical difficulties, or transfer conditions, the number of participants was not the same for the whole set of effect biomarkers. Thus, a subset of 284 to 299 individuals [191–215 workers exposed to Cr(VI) and 84–93 workers without known exposure to Cr(VI)] out of the 602 individuals who agreed to participate in the overall HBM4EU chromate study, were assessed for the selected effect biomarkers. Overall, 57 participants got results for the whole set of effect biomarkers. Concerning the analysis of MN PBL, 191 workers and 93 controls from 5 countries were included; MN RET were analyzed in 170 workers and 86 controls from all countries (except The Netherlands). The comet assay was performed in a subset of 74 workers and 43 controls from all countries (except The Netherlands) because it was limited to samples that were transferred within 24 h after collection and could be immediately processed. Oxidative stress biomarkers were measured in the urine samples from 215 workers and 84 controls from five countries.

Socio-demographic and lifestyle characteristics of participants and their distribution in exposed and control groups per effect biomarker are presented in Table 1. Overall, the great majority of the study participants were male (88%) of an average age of 42 years. They were predominantly non-smokers (53%) or former smokers (20%) and approximately the same proportion referred low (39%) or high (41%) alcohol consumption while 20% did not consume alcohol. The majority resided in urban areas (64%), with low (51%) to medium (39%) traffic density and with no industrial plants nearby (75%). Some differences were found between the workers and controls for the distribution of the participants according to gender and smoking status, irrespective of the effect biomarker that was analyzed. In general, the control group consistently included a higher proportion of women and a lower proportion of smokers compared with the exposed group (Table 1)

### 3.2. Activities at the Workplace and Exposure to Cr(VI)

**Figure 1:**
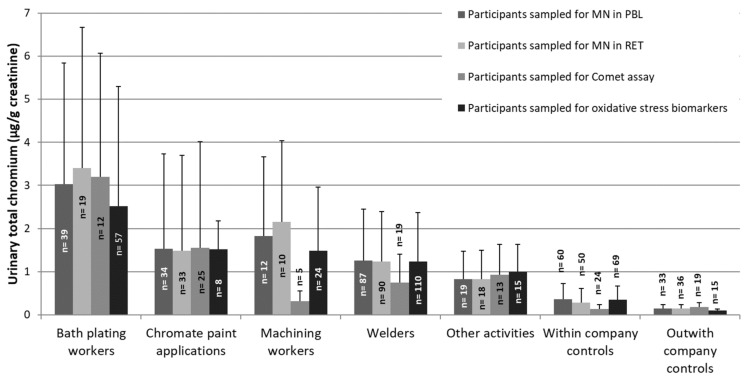
Mean (±SD) urinary total chromium levels in post-shift urine samples per exposure activity and control groups that were assessed for each of the different effect biomarkers.

Figure 1 shows the distribution of the U-Cr level that was measured at the end of the shift for the subsets of participants with results for the different effect biomarkers.

In general, each exposed subgroup displayed significantly higher mean U-Cr levels than the total control group, the within company or the outwith company control subgroups (*p* ≤ 0.007, MW test), irrespective of the effect biomarker being considered. Electrolytic bath platers showed the highest mean U-Cr level, being significantly higher than that of all the other exposed subgroups (*p* < 0.027), except the machining workers (*p* = 0.220). However, the small number (n = 5) of machining workers who provided samples for comet assay presented a very low level of U-Cr and this possibly biased the exposure level of this subgroup that did not reach statistical significance over the controls (*p* = 0.090).

No significant differences in the U-Cr levels were noted among welders, chromate painters, and workers that were involved in machining and those that were involved in other activities, irrespective of the effect biomarker that was considered. Regarding controls, significantly higher mean levels of U-Cr were measured in the within company controls compared with the outwith controls with data for MN PBLs and for oxidative stress biomarkers (p = 0.035 and *p* < 0.001, respectively). It must be noted that the outwith company control subgroup included only participants from Portugal and Finland (n = 33) regarding genotoxicity biomarkers and only from Finland (n = 15) regarding oxidative stress biomarkers (Table 1). This reasoning, together with the previous knowledge that “within company controls” displayed measurable levels of U-Cr [32] are behind the use not only of the total control group, but also of each of the two control subgroups for statistical evaluation.

### 3.3. Genotoxicity Biomarkers in Blood Cells

The results of the genotoxicity biomarkers per activity subgroup are depicted in Table 2.

Statistical evaluation of data that were obtained for the various parameters of the MN PBL assay (frequencies of MNBC, MN in BC, NPBs, NBUDs, and CBPI) did not reveal any statistically significant differences between the total exposed and the total control group. However, the comparison of the same parameters between the total exposed and the outwith company control groups showed that the exposed group displayed significantly increased mean frequencies of MNBC, MN in BC, NPBs (*p* = 0.033; *p* = 0.046; and *p* = 0.010, respectively). The CBPI values were also significantly higher in the exposed than in the outwith control subgroup (*p* = 0.012). The same trend was observed for the frequency of MN RET in the total exposed group that only differed significantly from that of the outwith company controls (*p* = 0.032). Concerning the comet assay results, the total exposed group showed a significantly increased level of DNA damage, inferred from the comet tail intensity, compared with both the total control and the outwith company control groups (*p* = 0.014 and *p* < 0.001, respectively) (Table 2).

In addition, statistically significant differences were observed between the within company control and the outwith company control subgroups for all the parameters that were analyzed (e.g., *p* < 0.001, *p* = 0.041 and *p* < 0.001 for MNBC, MN RET and DNA tail intensity, respectively). The outwith company control subgroup solely included participants from Portugal and Finland (n = 33 for MN PBL and n = 26 for MN RET). On the other hand, the Portuguese outwith company controls (n = 18) displayed significantly lower levels of MNBC/1000BC than the Finnish controls (n = 15) (*p* < 0.001) and the latter presented even higher levels of both effect biomarkers than the corresponding within-company controls (n = 10).

Following the disaggregation per activity performed, statistically significant differences were found for MN PBL, MN RET, and comet tail intensity among the subgroups of workers (*p* ≤ 0.001, KW test).

Pairwise statistical comparisons showed that workers that were involved in electrolytic bath plating, followed by chromate paint application, and other activities showed the highest frequencies of MNBC and MN in BC. A statistically significant increase in the mean MNBC frequency was observed for those subgroups compared with the outwith company control subgroup (*p* < 0.001, *p* = 0.041 and *p* = 0.035, respectively, MW U-test). Bath platers also exhibited the highest frequency of NPBs, while machining workers showed the highest NBUDs frequency (Table 2). The frequencies of NPB and NBUD were significantly increased in bath platers, painters, and other activities’ workers over those of the outwith company controls (*p* = 0.005 and *p* = 0.014, respectively). The lowest CBPI value was found in bath platers and the highest one in machining workers, suggesting opposite effects on the cell progression through the cell cycle.

Welders exhibited the lowest frequencies of MNBC and MN in BC. The frequency of MNBC was even significantly lower than that of the total control group (*p* = 0.005); the difference turned out to not be significant for the comparison with the outwith company controls (*p* = 0.582). Welders also presented significantly lower values of MNBC, when compared with workers that were performing bath plating (*p* < 0.001), chromate paint application (*p* = 0.045), and other activities (*p* = 0.042). However, welders displayed the highest MN RET frequency that was significantly increased over that of the total control (*p* < 0.001) and the outwith control groups (*p* < 0.001). Inversely, bath platers showed the lowest frequency of MN RET (Table 2). It must be noted, however, that MN RET and MN PBL were not quantified in exactly the same individuals, i.e., 19 bath platers (and 1 machining worker, all from Netherlands) were not analyzed for MN RET (Table 1). However, the exclusion of these individuals from the analysis of MN PBL (to allow a more direct comparison between MNBC and MN RET frequencies) decreased the mean frequency of MNBC that was measured for bath platers from 12.56 to 9.82, approaching that of chromate painters, without affecting the overall ranking based on the activities that were performed. Apart from welders, no other significant differences in MN RET were found between the remaining subgroups of workers and controls.

Regarding DNA damage evaluation by the comet assay, both bath plating workers and welders showed the highest levels, whereas the machining workers presented the lowest one. The level of DNA damage was significantly increased in welders over the total control group (*p =* 0.002). All workers’ subgroups differed significantly from the outwith company control subgroup (0.001 < *p* < 0.003).

### 3.4. Oxidative Stress Biomarkers in Urine

**Figure 2:**
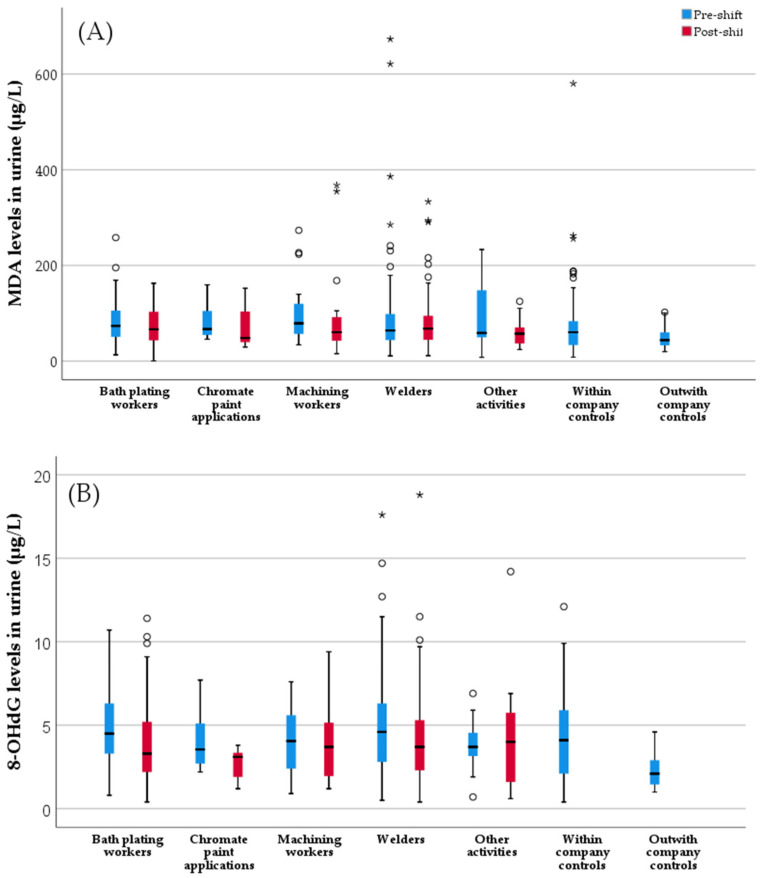
Levels of (**A**) malondialdehyde (MDA) and (**B**) of 8-hydroxydeoxyguanosine (8-OHdG) in pre- (in blue) and post-shift (in red) urine samples from groups of workers that were exposed to Cr(VI) and controls. * Extreme values; ^o^ outliers.

The results of MDA and 8-OHdG measured in pre- and post-shift urine samples from exposed workers and in a single urine sample from the controls are shown in Figure 2 (and Table S3). Comparison of pre-shift and post-shift MDA and 8-OHdG levels showed a trend for higher levels of both biomarkers in the pre-shift samples, although significant differences between the pre- and post-shift samples were only reached for 8-OHdG levels (*p* = 0.002).

The exposed group showed significantly higher MDA and 8-OHdG levels in the pre-shift samples compared with both the total control group (*p* = 0.002 and *p* = 0.008, respectively), and the outwith company control subgroup (*p* = 0.007 and *p* < 0.001, respectively). In the pre-shift samples the MDA level was also significantly raised in the exposed workers over the within company controls (*p* = 0.021). Following stratification of workers according to the activities performed, the MDA and 8-OHdG levels were significantly different among the workers subgroups (*p* = 0.031 and *p* = 0.004, respectively). All of the subgroups of workers showed significantly increased levels of MDA and 8-OHdG compared with the outwith company control subgroup (*p* ≤ 0.046 and *p* ≤ 0.01, respectively). The pre-shift MDA and 8-OHdG levels were also significantly higher in welders (*p* = 0.046 and *p* = 0.010, respectively) and in bath platers (*p* = 0.008 and *p* = 0.012, respectively) than in the total control group. The level of 8-OHdG was significantly raised in the within-company controls over the outwith company controls (*p* = 0.011). Concerning the results of post-shift samples, the 8-OHdG level of chromate paint applicators was significantly decreased compared with that of the within-company control group (*p* = 0.004). The other comparisons did not evidence significant differences between the exposed and the controls.

### 3.5. Effects of Predictor Variables on Genotoxicity and Oxidative Stress Biomarkers

The results of the different effect biomarkers were further explored in light of the information on socio-demographic and lifestyle factors, to assess the influence of the different predictor variables (e.g., country, age, gender, tobacco or alcohol consumption) on the dependent variables that were evaluated, i.e., MN PBL, MN RET, and comet tail intensity (Tables S1 and S2). The results of the KW analysis showed that, besides the country of origin that affected all genotoxicity biomarkers, also gender, age group, and alcohol consumption significantly affected the results of MN PBL when all the participants were considered. The MNBC/1000 BC frequency was significantly higher in women than in men (*p* = 0.004), in the group above 50 years than in the youngest group (*p* = 0.014), and in the high alcohol consumption subgroup compared to the medium and low consumption subgroups (*p* < 0.001). However, after participants’ categorization in the exposed and control groups (all controls or subdivided per within and outwith company controls) the effect of gender over the MN PBL and MN RET frequencies was only significant for the total control group (*p* = 0.001 and *p* = 0.041, respectively). On the other hand, the effect of age lost statistical significance, while alcohol consumption showed a significant influence on the level of MNBC/1000 BC in both the exposed, total control, and within-company control groups (*p* = 0.002, *p* = 0.007 and *p* = 0.040, respectively). Alcohol consumption also affected the frequency of MN RET, but uniquely in the total exposed group (*p* < 0.001). Inversely, tobacco smoking habits did not significantly influence the frequency of any of the effect biomarkers that were analyzed, except the frequency of MNBC/1000 BC in the outwith company control group (*p* = 0.006) with former smokers presenting the highest value (Tables S1 and S2). Residence location in an urban or rural area and the presence of industrial plants in the vicinity did not influence any of the dependent variables, and traffic intensity appeared to inversely affect the comet assay results only. Therefore, the potential influence of tobacco smoking, residence location, presence of industrial plants, and traffic intensity were not considered in further multiple regression analyses.

The influence of the U-Cr level on each effect biomarker was assessed by stratifying the participants by terciles of U-Cr level (Tables S1 and S2). When considering all the participants, the level of U-Cr showed a significant association with the MNBC frequency (7.90 ± 5.24, 8.54 ± 5.38, and 11.1 ± 7.09 for the 1st, 2nd, and 3rd terciles, respectively; *p* = 0.001). U-Cr similarly affected the MN RET frequency (2.65 ± 1.97, 3.33 ± 2.34, and 2.34 ± 1.40 for the 1st, 2nd, and 3rd terciles, respectively; *p* = 0.043) and the level of DNA damage (4.71 ± 2.89, 5.55 ± 2.35, and 6.76 ± 1.61 for the 1st, 2nd, and 3rd terciles, respectively; *p* = 0.002). After stratification into exposed and control groups, the differences among the 1st, 2nd, and 3rd terciles of U-Cr levels remained significant for MNBC/1000 BC and MN RET frequencies of the exposed group (*p* < 0.001 and *p* = 0.02), respectively. In the within-company controls, a trend for an association between the frequency of MNBC/1000BC and the level of U-Cr (10.3 ± 6.7, 12.3 ± 6.7, and 16.8 ± 7.5 for the 1st, 2nd, and 3rd terciles, respectively) was noted, although it did not reach statistical significance (*p* = 0.219).

Statistical evaluations between the exposed and control subgroups were further performed using a multiple linear regression model that included the most influencing variables (country, gender, alcohol consumption, and activity performed) on the levels of each effect biomarker that was studied. Regarding the total exposed group, the activity that was performed, and the country remained as variables significantly affecting the frequency of MNBC, after adjusting for gender and alcohol consumption, (*p* < 0.001; R^2^ = 0.35). As to the control group, the linear regression analysis evidenced that not only the country, but also gender, alcohol consumption, and the type of control significantly affected the MNBC frequency, after adjusting for the remaining variables (*p* = 0.004, *p* = 0.041, *p* = 0.017, and *p* = 0.030, respectively; R^2^ = 0.40).

The linear regression analysis was subsequently applied to assess the differences between the total exposed group or each of the activities’ subgroups and total controls or control subgroups, after adjusting for the effect of country, gender, and alcohol consumption. Concerning the MNBC/1000BC frequency, the results showed no statistically significant differences between the total exposed group and the total control group (*p* = 0.111; R^2^ = 0.36) or the outwith company control subgroup (*p* = 0.897; R^2^ = 0.36). The analysis also showed that the chromate painters were the only subgroup that displayed a significantly increased frequency of MNBC/1000 BC over both the total and outwith company control groups, after adjusting for the effect of those variables (*p* = 0.018; R^2^ = 0.24 and *p* = 0.046; R^2^ = 0.56, respectively).

With respect to MN RET, the frequency of the total exposed group was significantly higher than that of the outwith company control group (*p* = 0.004; R^2^ = 0.24), after adjusting for the effect of country, gender, and alcohol consumption. Using a similar assessment, no significant difference was detected between the total exposed and the total control groups (*p* = 0.181; R^2^ = 0.26). However, none of the activities’ subgroups displayed a significantly higher MN RET frequency compared to either the total control or the outwith company control group, after adjusting for the effect of the referred predictor variables.

The comparison of the level of DNA damage between the total exposed and the control groups or considering the activities’ subgroups showed significantly higher levels of DNA damage in bath platers, chrome painters and welders (*p* = 0.001, *p* < 0.001, *p* = 0.003; R^2^ = 0.59) compared with the total controls, after a similar adjustment as above. When each subgroup was compared with the outwith company control group, significant differences were detected for all the subgroups (*p* < 0.001 for all subgroups except for machining workers, *p* = 0.002; R^2^ = 0.74).

The only independent variable that significantly affected both oxidative stress biomarkers in the exposed group was the workers’ country (*p* < 0.001). Neither the different activities that were performed, nor the U-Cr level affected the oxidative stress biomarkers that were measured in urine, except the MDA level in pre-shift samples (*p* = 0.026) (Supplementary Tables S1 and S2). Although multiple regression models were explored to compare both biomarker levels between the exposed and control groups after adjusting for the effect of country, gender, and alcohol consumption, the assumptions of normality of the residuals and homoscedasticity of the residual variance were not met, indicating that these models were not reliably applicable.

### 3.6. Correlation between Effect Biomarkers

The results of the Spearman correlation analysis that was performed between the effect biomarkers are presented in Table 3.

Considering all the participants, the highest correlation was identified for the levels of MDA and 8-OHdG, either in the pre-shift or in post-shift urine samples (correlation coefficient = 0.585 and 0.529; *p* < 0.001). This correlation was also observed in the subgroups of exposed workers and controls, except for the correlation between MDA and 8-OHdG level in the post-shift urine samples that did not reach statistical significance (correlation coefficient = 0.435; *p* = 0.092). A statistically significant correlation was observed between the levels of DNA damage and MN PBL, considering all the participants or considering the total control group only (correlation coefficient = 0.626; *p* < 0.001); no correlation was found between the two biomarkers within the total exposed group (correlation coefficient = 0.206; *p* = 0.081). An inverse significant correlation was identified between the frequencies of MN PBL and MN RET for the total participants groups (Table 3) or the exposed group only (correlation coefficient = −0.256; *p* = 0.001).

### 3.7. Correlation between Effect and Exposure Biomarkers

The results of statistical analysis to explore correlations between exposure to Cr (assessed by plasma, red blood cells, or urine levels) and the results from each effect biomarker are presented in Table 4.

Although no strong correlations were found, the levels of MN PBL or DNA damage showed a correlation with the levels of Cr in the plasma and in the pre- and post-shift urine samples. Weak inverse correlations were detected between the MDA and 8-OHdG levels and U-Cr in post-shift urine samples.

## 4. Discussion

In the present study, biomarkers of genotoxicity and oxidative stress were analyzed in workers that were exposed to Cr(VI) spanning over three major industrial activities, i.e., electrolytic Cr(VI) plating in baths, other surface treatment activities, and welding. This subset of workers was part of a larger occupational study in which Cr(VI) and total Cr exposure had been characterized through blood and urine exposure biomarkers, besides airborne exposure and dermal contamination measurements, confirming different exposure levels according to the industrial sectors and activities that were performed [32].

The post-shift U-Cr level was selected to confirm this subset of participants’ exposure to Cr, given that it has previously shown a moderate to high correlation with both air Cr(VI) concentration and dermal total Cr contamination [32]. The main limitation of using U-Cr to evaluate Cr(VI) exposure is related to its non-specificity, because it measures Cr(VI) and Cr(III) and it is known that food can be a source of Cr(III) thus influencing U-Cr levels [32,61]. The U-Cr levels evidenced the higher exposure of workers that were involved in electrolytic bath plating compared to those that were involved in tasks that were related to surface treatment or welding. The welders’ subgroup displayed, in general, a slightly lower internal exposure level than that of chromate painters and machining workers but not reaching statistical significance. The variability of the U-Cr results (wide SD) within the same subgroup is likely to reflect interindividual variations in the population that was studied, comprising of, e.g., geographic, age, gender, diet, environmental exposure, and variables that are associated with the workplace. The variability is less likely to be related to interlaboratory variations given that all the laboratories that carried out the U-Cr analysis had previously passed an ICI within a QA/QC Program that was developed and implemented by the HBM4EU Initiative [52]. Overall, the present internal exposure levels agree with the results for the whole set of workers that were involved in the chromates study, that showed the highest internal exposure in bath platers, whereas welders presented the lowest one [32].

Regarding the control group, it cannot be ignored that the within-company controls are also exposed to low levels of Cr(VI), as shown in Figure 1. This observation had already been reported and discussed by Santonen et al. [32] for the overall chromate study, possibly reflecting an indirect exposure of those workers to Cr species in the occupational setting. Although environmental exposure to Cr(VI) occurs mainly through tobacco smoke or known or unknown environmental exposures [4], we observed no significant differences between the two control subgroups for smoking habits or living nearby industrial settings.

The results of the several effect biomarkers that were studied revealed that within-company controls displayed higher levels of DNA damage, chromosomal alterations, and oxidative stress (8-oxodG) than the outwith company controls. The fact that the latter subgroup solely included participants from Portugal and Finland may have introduced some bias in the reported levels of effect biomarkers. The levels of MN PBL and MN RET biomarkers that were estimated in the Portuguese controls are comparable to those that were previously reported in studies from the same country [36,54,62] favoring a geographical variation of the MN PBL frequency that may be related to different dietary habits or lifestyle factors. In addition, the MN PBL frequency of the outwith company control subgroup (mean MNBC = 7.33 ± 5.47/1000 BC; median = 5.50/1000 BC) is within the inter-quartile range of 3 and 12 MNBC per thousand BC and near the overall median MN frequency of 6.5 per thousand BC that was estimated for non-exposed subjects, based on data from approximately 7000 individuals that were gathered within the HUMN Project [63]. The within-company control MN PBL frequency (mean = 12.19 ± 7.58/1000 BC; median = 10.2/1000 BC) was higher than the median frequency that was reported by Fenech et al. [63]. Regarding the comet assay results that were obtained for this subgroup (Table 2), it slightly exceeded the interquartile interval of the tail intensity (between 1.1 and 6.7%) for control individuals [42] that was estimated in the scope of the hCOMET project that gathered a large dataset of subjects with comet assay results.

Overall, these findings reinforce the results of exposure biomarkers, highlighting that in Cr-related industries, office workers exposure to Cr (and, possibly to mixtures of Cr with other metals such as nickel and manganese) is associated with detectable genotoxic effects that may result in long-term adverse health outcomes. This may happen due to a false perception of safety and no adoption of protective measures by these workers. This evidence should not be neglected and justifies the need of these workers also being included in actions aiming to prevent occupational disease. On the other hand, although in occupational cross-sectional studies’ design the control group is frequently selected among “non-exposed” workers (commonly, office staff) from the same company (see e.g., [50]), from this study it is clear that the selection of the control group within the same company where workers are recruited constitutes a limitation that needs to be addressed in the design of future occupational studies.

Regarding of genotoxicity biomarkers that were analyzed in blood cells from exposed workers, the overall results showed an association between exposure and increased levels of DNA breaks (comet assay) and chromosomal alterations (MN assays), which are known to underlie cells malignant transformation.

The increased frequency of MN PBL is of particular relevance, due to its predictive value of cancer risk [34,64]. Of note, a positive correlation was shown between the levels of DNA and chromosomal damage in white blood cells in the groups of all the participants and all controls. Conversely, a poor inverse correlation was identified between the frequencies of MN PBL and MN RET. These findings contradict those of a previous study, where a significant positive correlation between the two biomarkers of chromosome damage was found in a Portuguese population that was exposed to pesticides [36]. A previous study suggested that a correlation between MN RET and MN PBL may be affected by the kinetics of the appearance of micronuclei and sampling schedule of the peripheral blood [40]. Indeed, the time window of the three genotoxicity biomarkers that were studied is quite different. The comet assay depicts DNA damage in total leukocytes (subpopulations with different life spans and DNA repair capacities) at the time of sampling. Due to the short life-span of RET, MN RET characterize recent genetic damage that has happened in bone marrow approximately three days prior to sample collection (i.e., in the beginning of the shift), as opposed to MN PBL which allows for the detection of accumulated DNA damage that is induced in lymphocytes for some time until sampling (depending on the repair of the lesions) and are capable of developing micronuclei during cells division in vitro. Another hypothesis to explain the discrepancy in MN frequency between PBL and RET might rely on eryptosis, induced by toxic effects of Cr(VI) via mitochondrial injury, DNA damage, increased cytosolic Ca2+ activity, and ATP depletion, leading to the loss of the most injured erythrocytes [65].The MN cytome assay allows for the detection of clastogenic and aneugenic events occurring during mitosis, besides the formation of nucleoplasmic bridges originating from chromosome rearrangements, e.g., dicentric chromosomes and events of DNA amplification that are seen as nuclear buds [55,56,63]. Although it is debatable whether MN in binucleated lymphocytes can reflect aneugenic effects that are induced in vivo, it is recognized that if the exposing agent is carried with the sample to the lymphocyte culture, MN might be induced through an aneugenic or clastogenic mechanism in vitro [43]. These results are in line with those of previous studies on occupational exposure to Cr(VI), consistently showing induction of MN in PBL [30,46,50,66,67] or in buccal cells [46]. Wang et al. (2012) described an association between decreased folate levels in chromium-exposed workers and an increased DNA damage that was measured by the comet assay, besides global DNA hypomethylation [68]. In a study by Sudha et al. (2010), welders showed a significant increase in micronucleated cells compared to controls [47]. Although in one study, stainless steel production workers did not show increased micronuclei frequency in nasal cells compared to the control group, it must be stressed that the control subjects were exposed to low chromium levels, as well [69]. In addition, Maeng et al. (2004) showed an increase in chromosomal aberrations (including chromatid exchanges and translocations, the latter was examined by fluorescent in situ hybridization) in chromium plating workers, which may reflect the cross-linking potential of chromium [70]. Moreover, some published occupational studies have also reported increased levels of DNA damage in leukocytes from exposed workers [30,71]. In addition, we observed that the CBPI value, which is not related to genotoxicity, was also significantly higher in the exposed than in the outwith controls. This observation may suggest that lymphocytes from exposed individuals have a higher proliferation rate in culture, although its relationship with a health outcome remains uncertain.

In this work, after categorization of participants per terciles of U-Cr level, direct associations between chromium internal exposure and the frequencies of MN PBL and MN RET or the level of DNA damage was observed. Those associations remained significant for the frequencies of MN PBL and MN RET in the total exposed group. The results of the correlation analysis further showed a correlation between the level of DNA or chromosomal damage in leukocytes and the levels of Cr in plasma and urine samples, suggesting that these effect biomarkers are sensitive to detect early biological effects that are elicited by low-level exposures.

Concerning the effect biomarkers in workers that were categorized per activity that was performed, the highest levels of chromosomal alterations in lymphocytes and of DNA damage in leukocytes were observed among bath platers, who also showed the highest internal exposure (measured by U-Cr). However, bath platers presented the lowest chromosomal damage level in reticulocytes. On the other hand, welders had the highest frequency of MN RET despite the lower U-Cr levels. It is known that similar Cr exposures through inhalation lead to approximately two-fold lower U-Cr levels in welders compared to Cr platers, suggesting a decreased Cr bioavailability that is present in welding fumes, which might explain the apparent discrepancy that was observed [32,72]. Due to the limited life-span of reticulocytes, the suitability of the MN RET assay for the detection of low-dose chronic exposures in occupational settings must be studied further. The assessment of MN RET [40] seems to be more sensitive to detect biological effects from recent exposure. The increased MN RET level in welders may also reflect exposure to the mixture of Cr with other genotoxic agents, e.g., nickel in welding fumes, even though this should have also been captured by the MN PBL and the comet assays.

The wide SD that affected, in general, the effect biomarkers data is not likely due to the potential influence of interlaboratory variations in analyses performance because this was minimised by centralizing the analysis of each endpoint in a single laboratory. Thus, the observed variability may be attributed to factors, e.g., age, gender, diet, smoking habits, or to exposure determinants in the occupational settings, besides individual susceptibility (e.g., polymorphisms of DNA repair genes), which was not investigated in this study. Several studies have shown that the MN PBL frequency is strongly affected by confounding variables such as gender and age and not so much affected by other, e.g., smoking habits [63]. In this study, gender was a confounding variable that influenced only the MN PBL and MN RET frequencies of the total control group. Among the controls, women displayed significantly higher frequencies of MN PBL than men; a similar gender-related trend was found for the exposed group, although not reaching statistical significance (Tables S1 and S2). This finding is in line with the estimation that women have, on average, a higher MN frequency compared to men [63]. After adjusting for the effect of country, gender, age, and alcohol consumption (which were the variables that affected this endpoint the most) using a multiple regression analysis model, only the group of chromate painters maintained a significantly increased level of MN PBL over the outwith company controls. Inversely, for the comet assay results, the previously identified differences between the exposed and control groups remained significant. This is in agreement with the work by Azqueta et al. (2020) on the confounding or intrinsic factors that may affect the comet assay results in HBM studies, showing that the comet assay results are independent of age, gender, or smoking habits of the study population [73].

These findings reinforce the relevance of matching exposed and control groups for confounding variables, to obtain reliable and consistent effect biomarker results. They also indicate that this is particularly relevant for the micronucleus assessment, which has clearly shown its value as an effect biomarker in numerous HBM studies. From this perspective, the comet assay seems to be more advantageous for application in multicentric studies because it is less affected by external variables that are hard to control.

The quantification of the pre-mutagenic 8-OHdG adduct has been considered as a valuable exposure and effect biomarker that is associated with an increased risk of cancer development [20,74]. In addition, the measurement of lipoperoxidation products, such as MDA, in plasma or urine of exposed individuals, has been commonly used as an effect biomarker in HBM studies regarding Cr(VI) exposure (reviewed in [5]). The characterization of oxidative stress biomarkers in urine also showed significantly increased levels in exposed workers and in each activity subgroup compared to controls, reinforcing the existence of early effects that were related to Cr(VI) exposure. Some authors have shown high inter- and intra-individual variabilities for 8-OHdG, ranging from 50% to 75% in spot urine samples that could be partially compensated by creatinine normalization [75,76]. Thus, the high variability that was found in 8-OHdG and MDA concentrations might be related to the lack of results adjustment to creatinine values. As can be seen in Figure 2, workers displayed higher levels of MDA and 8-OHdG in the pre-shift compared to the post-shift urine samples. Although this finding is unexpected, the different stages of elimination of Cr that has been accumulated throughout the working week, may help explaining it. For example, in welders a two- or three-stage process of elimination with half-lives of 7 h, 15–30 days, and 3–5 years has been suggested [15]. In chrome platers, Cr half-lives of 2–3 days followed by 1 month have been shown [77]. Thus, although occupational Cr exposure ceases during the weekend, a part of the absorbed Cr remains in the body while it is slowly excreted and may continuously generate oxidative stress. In addition, the contribution of other factors, e.g., food-related, cannot be excluded.

A good correlation between MDA and 8-oxodG levels was evidenced both in the pre- and post-shift urine samples. Our results agree with those that were previously reported in other studies [78,79] for example, in chrome electroplating workers. Pan et al. (2018) also identified an association between Cr exposure and the urinary measures of MDA and 8-oxodG [78]. However, other authors observed no statistically significant correlation between the MDA and blood or urinary Cr levels [70]. This lack of correlation might be explained by the time of sampling as it has been shown in animal models that the generation of ROS is detected during the earlier phase of exposure only, and that levels return to the control level afterwards [80].

Despite the advantage of gathering data and knowledge on the early biological effects from exposure to Cr(VI) in workers from several industries and activities, the study has also some limitations that may have had an effect on the results that are presented. Regarding the focus on Cr or Cr(VI), there was no opportunity to study other work-related factors in depth. Other co-exposure, e.g., to heavy metals and their combined effects may also in part explain some of the observed effect biomarker results. Although the procedures were well harmonized, it is possible that some of the differences that were observed between countries are the result of differences in the application of study protocols, besides the factors that have been already discussed. A potential selection bias in the process of recruitment of companies due to regulatory and societal context that may differ from country to country may have also influenced the decision to participate or not participate. For some effect biomarkers, the study sample was incomplete, which resulted in a lower number than anticipated, thus reducing the statistical power, particularly, after categorization of workers per activity [32]. Some of the most interesting comparisons are those between the workers and the controls. Although a second group of (outwith) controls was included, this group was available only in two participating countries which is a limitation regarding the observed differences between countries for some of our study parameters.

## 5. Conclusions

In the present study, the effect biomarkers contributed to the interpretation of exposure biomarker data and went beyond by assisting in the identification of groups at risk that had not been captured by exposure biomarkers analysis. This was the case of welders that despite displaying the lowest internal exposure level (1.26 ± 1.19 µg/g creatinine, assessed by urinary Cr levels) revealed the induction of chromosomal alterations (3.4 ± 2.3 MN RET) in their blood.

Another paradigmatic example was the evidence that the controls that were recruited among administrative staff of the industries that were involved in the study displayed levels of genetic alterations in blood cells and oxidative damage markers in urine that were higher than expected for a “control” group. This finding not only supports their exposure to low Cr(VI) level but also suggests that this type of exposure has deleterious biological effects and thereby can result in disease development. Although further research is needed with a higher number of office workers from these industries in order to confirm this finding and to ascertain the factors that may explain it, intervention measures should be put in place to protect these workers’ health.

Our findings of significantly increased genome damage and oxidative stress that were detected in the studied workers and, particularly in the subgroups of chrome painters and electrolytic bath platers, who showed the highest level of genetic damage in blood lymphocytes (9.72 ± 6.36 and 12.56 ± 8.04, respectively), suggest that Cr(VI) exposure might still represent a health risk, even though the exposure levels were mostly well below the current binding OEL in EU. Thus, this study reinforces the need for further re-evaluation of the occupational exposure limit, better application of protection measures, and education of workers. On the other hand, it also raised some additional questions and unexplained inconsistencies that need follow-up studies to be clarified.

The correlation that was found between the frequency of DNA and chromosomal damage (assessed by the comet assay and MN assay in blood cells) and the levels of Cr in the plasma and in urine samples shows the value of those effect biomarkers, supported by mechanistic knowledge, to relate to exposure and health outcomes, such as cancer. This knowledge is expected to reinforce risk management measures leading to a better protection of workers.

## Figures and Tables

**Table 1:** Participants’ distribution among the exposure groups (workers/controls) according to their main characteristics.

Independent Variables	Participants with MN PBL Data n (%)			Participants with MN RET Data n (%)			Participants with Comet Assay Data n (%)			Participants with Oxidative stress Biomarkers Data n (%)		
	Total Participants	Total Workers	Total Controls ^a^	Total Participants	Total Workers	Total Controls ^a^	Total Participants	Total Workers	Total Controls ^a^	Total Participants	Total Workers	Total Controls ^a^
**n**	284	191 (67.3)	93 (32.7)	256	170 (66.4)	86 (33.6)	117	74 (63.2)	43 (36.8)	299	215 (71.9)	84 (28.1)
**Country**	284			256			117			299		
**Belgium**	69 (24.3)	48 (25.1)	21 (22.6)	69 (27.0)	48 (28.2)	21 (24.4)	29 (24.8)	16 (21.6)	13 (30.2)	67 (22.4)	53 (24.7)	14 (16.7)
**Finland**	56 (19.7)	33 (17.3)	23 (24.7)	61 (23.8)	37 (21.8)	24 (27.9)	20 (17.1)	14 (18.9)	6 (14.0)	72 (24.1)	47 (21.9)	25 (29.8)
**Netherlands**	30 (10.6)	20 (10.5)	10 (10.8)							30 (10.0)	20 (9.3)	10 (11.9)
**Poland**	71 (25.0)	52 (27.2)	19 (20.4)	69 (27.0)	51 (30.0)	18 (20.9)	8 (6.8)	5 (6.8)	3 (7.0)	71 (23.7)	52 (24.2)	19 (22.6)
**Portugal**	58 (20.4)	38 (19.9)	20 (21.5)	57 (22.3)	34 (20.0)	23 (26.7)	60 (51.3)	39 (52.7)	21 (48.8)			
**France**										59 (19.7)	43 (20.0)	16 (19.0)
**Sex**	284			256			117			299		
**Female**	35 (12.3)	7 (3.7)	28 (30.1)	30 (11.7)	7 (4.1)	23 (26.7)	15 (12.8)	5 (6.8)	10 (23.3)	32 (10.7)	4 (1.9)	28 (33.3)
**Male**	249 (87.7)	184 (96.3)	65 (69.9)	226 (88.3)	163 (95.9)	63 (73.3)	102 (87.2)	69 (93.2)	33 (76.7)	267 (89.3)	211 (98.1)	56 (66.7)
**Age**												
**Mean**	42.0	41.1	43.6	41.9	41.1	43.5	43.5	42.8	44.6	41.7	41.1	43.4
**SD**	10.4	11.0	8.88	10.5	11.1	9.28	9.94	11.0	7.89	10.5	10.6	9.88
**Min–max**	20–68	20–68	23–63	20–68	20–68	23–63	20–64	20–64	30–60	20–68	20–68	23–63
**Age group**	274			246			110			293		
**20–49**	200 (73.0)	132 (72.5)	68 (73.9)	179 (72.8)	116 (72.0)	63 (74.1)	76 (69.1)	47 (69.1)	29 (69.0)	217 (74.1)	154 (73.3)	63 (75.9)
**50–68**	74 (27.0)	50 (27.5)	24 (26.1)	67 (27.2)	45 (28.0)	22 (25.9)	34 (30.9)	21 (30.9)	13 (31.0)	76 (25.9)	56 (26.7)	20 (24.1)
**Smoking**	279			251			112			296		
**Smoker**	78 (28.0)	69 (36.9)	12 (13.0)	66 (26.3)	55 (33.1)	11 (12.9)	29 (25.9)	22 (31.4)	7 (16.7)	81 (27.4)	72 (33.8)	9 (10.8)
**Former smoker**	35 (12.5)	46 (24.6)	13 (14.1)	52 (20.7)	41 (24.7)	11 (12.9)	24 (21.4)	18 (25.7)	6 (14.3)	73 (24.7)	59 (27.7)	14 (16.9)
**Non smoker**	166 (59.5)	72 (38.5)	67 (72.8)	133 (53.0)	70 (42.2)	63 (74.1)	59 (52.7)	30 (42.9)	29 (69.0)	142 (48.0)	82 (38.5)	60 (72.3)
**Alcohol**	279			251			112			296		
**No**	51 (18.3)	34 (18.2)	17 (18.5)	45 (17.9)	29 (17.5)	16 (18.8)	30 (26.8)	21 (30.0)	9 (21.4)	40 (13.5)	29 (13.6)	11 (13.3)
**Low**	114 (40.9)	79 (42.2)	35 (38.0)	116 (46.2)	79 (47.6)	37 (43.5)	30 (26.8)	13 (18.6)	17 (40.5)	128 (43.2)	94 (44.1)	34 (41.0)
**High**	114 (40.9)	74 (36.9)	40 (43.5)	90 (35.9)	58 (34.9)	32 (37.6)	52 (46.4)	36 (51.4)	16 (38.1)	128 (43.2)	90 (42.3)	38 (45.8)
**Home location**	279			251			112			293		
**Urban**	199 (71.3)	130 (69.5)	69 (75.0)	175 (69.7)	113 (68.1)	62 (72.9)	84 (75.0)	55 (78.6)	29 (69.0)	187 (63.8)	127 (60.2)	60 (73.2)
**Rural**	80 (28.7)	57 (30.5)	23 (25.0)	76 (30.3)	53 (31.9)	23 (27.1)	28 (25.0)	15 (21.4)	13 (31.0)	106 (36.2)	84 (39.8)	22 (26.8)
**Industrial area**	275			247			112			294		
**No**	206 (74.9)	134 (73.2)	72 (78.3)	183 (74.1)	116 (71.6)	67 (78.8)	89 (79.5)	54 (77.1)	35 (83.3)	207 (70.4)	152 (72.0)	55 (66.3)
**Yes**	69 (25.1)	49 (26.8)	20 (21.7)	64 (25.9)	46 (28.4)	18 (21.2)	23 (20.5)	16 (22.9)	7 (16.7)	87 (29.6)	59 (28.0)	28 (33.7)
**Traffic density**	279			251			109			296		
**Low**	139 (49.8)	96 (51.3)	43 (46.7)	119 (47.4)	82 (49.4)	37 (43.5)	59 (52.7)	37 (52.9)	22 (52.4)	160 (54.1)	119 (55.9)	41 (49.4)
**Medium**	113 (40.5)	70 (37.4)	43 (46.7)	108 (43.0)	66 (39.8)	42 (49.4)	42 (37.5)	25 (35.7)	17 (40.5)	103 (34.8)	68 (31.9)	35 (42.2)
**Heavy**	27 (9.7)	21 (11.2)	6 (6.5)	24 (9.6)	18 (10.8)	6 (7.1)	11 (9.8)	8 (11.4)	3 (7.1)	33 (11.1)	26 (12.2)	7 (8.4)

^a^ Total controls include participants that were recruited within the company and outwith the company; MN: micronuclei; PBL: peripheral blood lymphocytes; RET: reticulocytes.

**Table 2:** Results of genotoxicity biomarkers that were analyzed in the blood cells from workers that were exposed to Cr(VI) and the control groups (mean ± SD).

	MN PBL						MN RET		Comet Assay	
	n	MNBC (‰)	MN in BC (‰)	NPB (‰)	NBUD (‰)	CBPI	n	Micronucleated +CD71 Reticulocytes (‰)	n	Tail Intensity (%)
**Total exposed Group**	**191**	9.11 ± 6.08 * ^£^	10.47 ± 7.19 * ^£^	**1.76 ± 2.92 ^£^**	0.57 ± 0.97 *	1.85 ± 0.26 * ^£^	**170**	**2.75 ± 1.92 ^£^**	**74**	6.34 ± 1.83 * ^£ ¥^
Bath plating workers	39	12.56 ± 8.04 ^£^	14.32 ± 9.64 ^£^	3.02 ± 3.56 ^£^	0.65 ± 0.98 ^£^	1.72 ± 0.33 *	19	1.88 ± 1.14 *	12	7.36 ± 1.61 ^£ ¥^
Chromate paint applicators	34	9.72 ± 6.36 ^£^	10.82 ± 7.07 ^£^	1.65 ± 2.98	0.40 ± 0.74 * ^¥^	1.83 ± 0.24	33	2.03 ± 1.11 *	25	5.24 ± 1.26 * ^£^
Welders	87	7.37 ± 4.78 * ^¥^	8.55 ± 5.79 * ^¥^	1.51 ± 2.89 ^£^	0.60 ± 1.08 *	1.86 ± 0.23 ^£^	90	3.40 ± 2.25 ^£ ¥^	19	7.62 ± 1.92 ^£ ¥^
Machining workers	12	8.04 ± 3.09	9.62 ± 4.01	0.63 ± 0.91 ^£^	0.79 ± 0.99 * ^¥^	2.00 ± 0.23 ^£^	10	2.30 ± 1.34	5	5.04 ± 2.03 ^£^
Other activities	19	9.60 ± 4.76 ^£^	11.27 ± 5.97 ^£^	1.17 ± 1.21	0.39 ± 0.74	1.98 ± 0.14 ^£^	18	1.97 ± 0.72	13	6.12 ± 1.04 * ^£^
**Total control Group**	**93**	**10.47 ± 7.26**	**11.88 ± 8.19**	**1.77 ± 2.36**	**0.48 ± 0.88**	**1.83 ± 0.29**	**86**	**2.62 ± 2.16**	**43**	**4.59 ± 3.26**
Within company	60	12.19 ± 7.58 ^£^	13.68 ± 8.40 ^£^	2.03 ± 2.36 ^£^	0.65 ± 0.97 ^£^	1.88 ± 0.30 ^£^	50	3.13 ± 2.67 ^£^	24	6.88 ± 2.44 ^£^
Outwith company	33	7.33 ± 5.47 *	8.61 ± 6.77*	1.29 ± 2.30 *	0.18 ± 0.41 *	1.74 ± 0.24 *	36	1.92 ± 0.68 *	19	1.71 ± 1.18 *

MN PBL—cytokinesis-block micronucleus assay in peripheral blood lymphocytes; MNBC—frequency of micronucleated binucleated cells per 1000 binucleated cells; MN—micronuclei per 1000 binucleated cells; NPB—nucleoplasmic bridges per 1000 binucleated cells; NBUD—nuclear buds per 1000 binucleated cells CBPI—cytokinesis-block proliferation index; MN +CD71 RET—frequency of micronucleated +CD71 reticulocytes (per 1000 +CD71 reticulocytes); MN RET—micronucleus assay in reticulocytes; PBL—peripheral blood lymphocytes; * Significantly different from the within company controls; ^£^ Significantly different from the outwith company controls; ^¥^ Significantly different from the total controls.

**Table 3:** Correlation between the effect biomarkers considering the data from all the participants.

		MN PBL	MN RET	Comet—Tail Intensity	MDA Pre-Shift	MDA Post-Shift	8-OHdG Pre-Shift	8-OHdG Post-Shift
MN PBL	n		247	**114**	307	162	207	162
	Corr. Coef		−0.143	**0.440**	0.042	0.070	0.090	0.022
	*p*		0.024	**<0.001**	0.547	0.376	0.197	0.786
MN RET	n			112	182	137	182	137
	Corr. Coef			0.078	−0.020	0.080	−0.069	0.096
	*p*			0.414	0.788	0.353	0.358	0.264
Comet—tail intensity	n				44	36	44	36
	Corr. Coef				−0.105	−0.025	−0.058	0.077
	*p*				0.499	0.885	0.710	0.653
MDA pre-shift	n					**228**	**298**	228
	Corr. Coef					**0.277**	**0.585**	0.114
	*p*					**<0.001**	**<0.001**	0.086
MDA post-shift	n						**228**	**229**
	Corr. Coef						**0.204**	**0.529**
	*p*						**0.002**	**<0.001**
8-OHdG pre-shift	n							**228**
	Corr. Coef							**0.335**
	*p*							**<0.001**
8-OHdG post-shift	n							
	Corr. Coef							
	*p*							

Strength of correlation according to the correlation coefficient (corr. Coef.) value: light orange cell: 0.1 ≤ 0.2 = poor; orange cell: 0.2 ≤ 0.5 = fair; light green: 0.5 ≤ 0.7 = moderate; light grey cell: tested, but no significant correlation found. MDA—malondialdehyde; 8-OHdG—8-hydroxy-2′-deoxyguanosine; MN PBL—frequency of micronucleated binucleated cells per 1000 binucleated cells; MN RET—frequency of micronucleated reticulocytes per 1000 +CD71 reticulocytes.

**Table 4:** Results of the correlation analysis between exposure and effect biomarkers.

		MN PBL	MN RET	Comet—Tail Intensity	MDA Pre-Shift	MDA Post-Shift	8-OHdG Pre-Shift	8-OHdG Post-Shift
Cr in plasma	n	**279**	251	**112**	284	217	284	217
	Corr. Coef	**0.361**	−0.038	**0.476**	0.068	−0.115	−0.033	−0.099
	*p*	**<0.001**	0.552	**<0.001**	0.256	0.091	0.576	0.147
Cr in red blood cells	n	279	251	**112**	284	217	**284**	217
	Corr. Coef	0.086	−0.098	**−0.216**	0.027	−0.020	**−0.126**	−0.063
	*p*	0.153	0.122	**0.022**	0.653	0.771	**0.035**	0.357
U-Cr (pre-shift)	n	**260**	233	**100**	283	219	283	219
	Corr. Coef	**0.175**	0.023	**0.303**	0.029	−0.074	0.067	−0.008
	*p*	**0.005**	0.723	**0.002**	0.626	0.273	0.258	0.910
U-Cr (post-shift)	n	**252**	224	**98**	277	**216**	277	**216**
	Corr. Coef	**0.207**	−0.009	**0.371**	0.079	**−0.207**	0.072	**−0.162**
	*p*	**0.001**	0.888	**<0.001**	0.190	**0.002**	0.234	**0.018**

Strength of correlation according to the correlation coefficient (corr. Coef.) value: 0.1 ≤ 0.2 = poor; 0.2 ≤ 0.5 = fair; 0.5 ≤ 0.7 = moderate. U-Cr—concentration of Cr in urine; MDA—malondialdehyde; 8-OHdG—8-hydroxy-2′-deoxyguanosine; MN PBL—frequency of micronucleated binucleated cells per 1000 binucleated cells; MN RET—frequency of micronucleated reticulocytes per 1000 +CD71 reticulocytes.

## Data Availability

Data supporting the reported results are available upon request to corresponding author.

## Supplementary Materials

The following supporting information can be downloaded at: https://www.mdpi.com/article/10.3390/toxics10080483/s1, Table S1: Mean frequencies (±SD) of micronucleated binucleated cells (MNBC), micronuclei in reticulocytes (MN RET), tail intensity (comet assay), and urinary levels of malondialdehyde (MDA) and 8-hydroxydeoxyguanosine (8-OHdG) in the exposed and control groups, after stratification according to socio-demographic, lifestyle, and occupational activity characteristics.; Table S2: Mean frequencies (±SD) of micronucleated binucleated cells (MNBC), micronuclei in reticulocytes (MN RET), tail intensity (comet assay), and urinary levels of malondialdehyde (MDA) and 8-hydroxydeoxyguanosine (8-OHdG) in control subgroups, after stratification according to socio-demographic, lifestyle, and occupational activity characteristics. Table S3: Level of oxidative stress biomarkers parameters (mean ± SD) in groups of workers exposed to Cr(VI) and controls.

## Appendix A

The HBM4EU Chromates study team consists of: Catarina Afonso ^1^ (methodology; investigation), Rob Anzion ^2^ (methodology), Radia Bousoumah ^3^ (investigation), Maurice van Dael ^3^ (methodology), Ogier Hanser ^3^ (methodology), Mira Hartikainen ^4^ (methodology), Katrien Poels ^5^ (methodology), Simo P. Porras ^4^ (methodology), Riitta Velin ^4^ (methodology) and Mariana F. Fernandez ^6,7,8^ (investigation); the correspondent affiliations are: ^1^ National Institute of Health Dr. Ricardo Jorge, Department of Human Genetics, Lisbon, Portugal; ^2^ Radboud Institute for Health Sciences, Radboudumc, Nijmegen, The Netherlands; ^3^ French National Research and Safety Institute, Vandœuvre-lès-Nancy, France; ^4^ Finnish Institute of Occupational Health, Helsinki, Finland; ^5^ and the Centre for Environment and Health, Department of Public Health and Primary Care, KU Leuven (University of Leuven), O&N 5b, Herestraat 49, box 952, 3000 Leuven, Belgium; ^6^ Centre of Biomedical Research (CIBM), University of Granada, 18016 Granada, Spain; ^7^ Biosanitary Research Institute of Granada (ibs.GRANADA), 18012 Granada, Spain; ^8^ CIBER de Epidemiología y Salud Pública (CIBERESP), 28029 Madrid, Spain.
