# Supplementary material for: HBM4EU Chromates Study—Genotoxicity and Oxidative Stress Biomarkers in Workers Exposed to Hexavalent Chromium
Source: Toxics. 2022 Aug 18;10(8):483. doi: 10.3390/toxics10080483 (PMC9412464; doi:10.3390/toxics10080483)
Supplement: Supplementary file 1 [file toxics-10-00483-s001.zip › toxics-1818379-supplementary.pdf]

Table S1. Mean frequencies (±SD) of micronucleated binucleated cells (MNBC), micronuclei in reticulocytes (MN RET), tail intensity (comet assay) and urinary levels of malondialdehyde (MDA) and 8-hydroxydeoxyguanosine (8-OHdG) in exposed and control groups, after stratification according to socio-demographic, lifestyle and occupational activity characteristics.

Independent variables	Exposed group					Total control group				
	MNBC (‰)	MN RET (‰)	Tail intensity (%)	MDA ((µg/)	8-OHdG ((µg/L)	MNBC (‰)	MN RET (‰)	Tail intensity (%)	MDA ((µg/L)	8-OHdG ((µg/L)
Country										
Belgium	11.3 ± 6.0	2.2 ± 1.1	7.0 ± 1.1	65.1 ± 36.5	3.4 ± 1.6	14.1 ± 7.1	2.9 ± 1.8	7.6 ± 0.9	55.4 ± 32.9	3.9 ± 2.2
Finland	9.4 ± 4.9	2.4 ± 1.6	8.2 ± 1.7	54.8 ± 34.1	2.8 ± 1.9	10.6 ± 5.6	2.1 ± 0.7		51.4 ± 26.3	2.4 ± 1.2
Netherlands	15.8 ± 8.9			76.7 ± 37.4	4.1 ± 2.7	17.0 ± 6.4			80.1 ± 82.3	5.2 ± 2.1
Poland	6.1 ± 3.7	4.1 ± 2.4	6.3 ± 1.1	85.3 ± 46.8	5.2 ± 4.6	9.1 ± 6.0	4.3 ± 3.1	7.5 ± 1.2	90.5 ± 98.1	4.8 ± 2.3
Portugal	6.6 ± 2.4	1.9 ± 1.0	5.5 ± 1.8			4.1 ± 1.7	1.7 ± 0.5	1.7 ± 1.2		
France				103.9 ± 82.1	4.4 ± 2.3				99.4 ± 79.1	4.9 ± 3.2
p	< 0.001	< 0.001	< 0.001	< 0.001	< 0.001	< 0.001	0.009	< 0.001	0.192	0.003
Gender										
Female	12.0 ± 6.8	3.3 ± 2.2	5.3 ± 1.1	84.8 ± 67.9	3.5 ± 0.6	12.2 ± 6.5	3.3 ± 2.8	4.1 ± 3.5	57.1 ± 58.8	4.0 ± 2.1
Male	9.2 ± 6.1	2.7 ± 1.9	6.5 ± 1.9	76.4 ± 52.9	4.0 ± 2.3	8.9 ± 6.5	2.4 ± 1.7	3.1 ± 2.8	81.6 ± 73.1	4.1 ± 2.6
p	0.452	0.736	0.171	0.800	0.510	0.010	0.041	0.196	0.001	0.257
Age										
20-49	8.9 ± 6.2	2.6 ± 1.7	6.3 ± 2.0	78.7 ± 57.7	4.0 ± 2.4	8.9 ± 6.2	2.6 ± 2.1	3.5 ± 3.0	75.2 ± 74.7	4.3 ± 2.6
50-68	10.1 ± 5.7	3.1 ± 2.3	6.8 ± 1.8	70.9 ± 37.8	4.0 ± 1.9	12.5 ± 7.4	2.5 ± 1.6	2.3 ± 2.6	71.5 ± 54.6	3.3 ± 1.7
p	0.099	0.649	0.343	0.928	0.505	0.078	0.413	0.707	0.437	0.428

Smoking status										
Smoker	9.4 ± 7.0	2.8 ± 1.4	5.8 ± 1.8	83.8 ± 60.2	4.2 ± 2.6	7.4 ± 6.7	2.1 ± 1.0	4.2 ± 3.0	92.4 ± 84.0	5.1 ± 2.5
Former smoker	8.9 ± 5.2	2.7 ± 2.1	6.5 ± 1.3	80.4 ± 63.2	3.9 ± 2.3	11.0 ± 6.8	2.5 0.9	2.5 ± 4.0	81.9 ± 69.2	3.7 ± 2.7
Non smoker	9.4 ± 5.8	2.7 ± 2.1	6.8 ± 2.2	67.8 ± 35.4	3.9 ± 2.0	10.0 ± 6.5	2.7 ± 2.3	3.1 ± 2.7	69.7 ± 68.1	4.0 ± 2.4
p	0.519	0.435	0.192	0.179	0.706	0.252	0.646	0.660	0.406	0.156
Alcohol consumption										
Low	8.7 ± 5.6	2.5 ± 1.5	6.3 ± 1.9	72.1 ± 38.9	3.6 ± 2.0	8.0 ± 5.7	2.3 ± 1.5	3.4 ± 3.3	58.6 ± 68.3	3.0 ± 1.9
Medium	8.23 ± 6.6	3.5 ± 2.2	6.7 ± 2.0	82.4 ± 53.0	4.4 ± 2.3	7.9 ± 5.4	3.0 ± 2.5	3.6 ± 3.0	79.5 ± 79.8	4.5 ± 2.3
High	10.6 ± 5.6	1.9 ± 1.0	6.5 ± 1.9	72.1 ± 56.5	3.8 ± 2.2	12.5 ± 7.4	2.1 ± 1.2	2.3 ± 2.6	74.7 ± 62.0	4.0 ± 2.6
p	0.002	<0.001	0.733	0.272	0.382	0.007	0.295	0.174	0.307	0.516
U-Cr end-shift (μg/g creatinine)										
1st tertile	7.0 ± 3.5	2.8 ± 2.0	6.1 ± 2.3	94.6 ± 71.5	4.3 ± 2.5	8.8 ± 6.2	2.5 ± 2.0	3.3 ± 3.0	75.1 ± 73.6	4.1 ± 2.5
2nd tertile	7.6 ± 4.2	3.6 ± 2.5	6.3 ± 1.8	81.3 ± 52.2	4.3 ± 2.4	11.7 ± 6.9	2.8 ± 1.9	2.6 ± 1.7	72.8 ± 62.9	4.1 ± 2.3
3rd tertile	11.0 ± 7.1	2.3 ± 1.3	6.9 ± 1.6	67.7 ± 44.0	3.7 ± 2.1	16.8 ± 7.5			69.4 ± 58.4	3.2 ± 1.8
p	<0.001	0.020	0.071	0.026	0.354	0.087	0.249	0.589	0.969	0.796
Activity/Group										
Bath plating workers	12.6 ± 8.0	1.9 ± 1.1	7.4 ± 1.6	72.67 ± 39.6	4.1 ± 2.6					
Chromate paint applicators	9.7 ± 6.4	2.0 ± 1.1	5.2 ± 1.3	70.6 ± 44.0	2.7 ± 0.9					

Welders	7.4 ± 4.8	3.4 ± 2.3	7.6 ± 1.9	78.6 ± 54.8	3.7 ± 2.0					
Machining workers	8.0 ± 3.1	2.3 ± 1.3	5.0 ± 2.0	88.8 ± 92.7	4.2 ± 2.7					
Other activities	9.6 ± 4.8	2.0 ± 0.7	6.1 ± 1.0	59.7 ± 30.5	4.1 ± 3.5					
Within company control						12.2 ± 7.6	3.1 ± 2.7	6.9 ± 2.4	74.1 ± 74.2	4.2 ± 2.6
Outwith company control						7.3 ± 5.5	1.9 ± 0.7	1.7 ± 1.2	51.0 ± 26.1	2.3 ± 1.1
p	0.001	< 0.001	< 0.001	0.745	0.608	0.001	0.041	< 0.001	0.368	0.013

Table S2. Mean frequencies (±SD) of micronucleated binucleated cells (MNBC), micronuclei in reticulocytes (MN RET), tail intensity (comet assay) and urinary levels of malondialdehyde (MDA) and 8-hydroxydeoxyguanosine (8-OHdG) in control subgroups, after stratification according to socio-demographic, lifestyle and occupational activity characteristics.

Independent variables	Within company control subgroup					Outwith company control subgroup				
	MNBC (‰)	MN RET (‰)	Tail intensity (%)	MDA (µg/L)	8-OhdG (µg/L)	MNBC (‰)	MN RET (‰)	Tail intensity (%)	MDA (µg/L)	8-OhdG (µg/L)
Country										
Belgium	14.1 ± 7.1	2.9 ± 1.8		55.4 ± 32.9	3.9 ± 2.2					
Finland	9.1 ± 4.8	1.8 ± 0.5	7.6 ± 0.9	52.2 ± 28.9	2.6 ± 1.5	11.1 ± 6.0	2.2 ± 0.8		51.0 ± 26.1	2.3 ± 1.1
Netherlands	17.0 ± 6.4		7.5 ± 1.2	80.1 ± 82.3	5.2 ± 2.1					
Poland	9.1 ± 6.0	4.3 ± 3.1		90.5 ± 98.1	4.8 ± 2.3					
Portugal						4.1 ± 1.8	1.7 ± 0.5	1.7 ± 1.2		
France				99.4 ± 79.1	4.9 ± 3.2					
p	0.001	0.175	0.144	0.293	0.049	< 0.001	0.040			
Gender										
Female	12.5 ± 6.6	3.7 ± 3.0	7.1 ± 1.0	57.7 ± 60.2	4.1 ± 2.2	10.7 ± 7.6	1.9 ± 0.8	1.1 ± 0.3		
Male	11.1 ± 7.1	3.2 ± 2.5	6.5 ± 3.1	92.1 ± 81.1	4.7 ± 2.6	7.1 ± 5.3	1.9 ± 0.7	1.8 ± 1.3	51.5 ± 27.0	2.3 ± 1.1
p	0.100	0.174	0.216	<0.001	0.067	0.408	0.687	0.219		
Age										
20-49	10.4 ± 6.4	3.3 ± 2.7	6.5 ± 2.9	82.0 ± 80.9	4.8 ± 2.6	7.0 ± 5.5	1.9 ± 0.6	1.9 ± 1.4	46.8 ± 25.6	2.2 ± 1.1
50-68	15.8 ± 7.2	3.7 ± 2.1	A	73.8 ± 60.0	3.5 ± 1.8	8.7 ± 5.9	1.9 ± 0.9	1.2 ± 0.5	62.5 ± 27.3	2.7 ± 1.2
p	0.093	0.561	0.897	0.623	0.219	0.462	0.596	0.517	0.240	0.433

Smoking status										
Smoker	12.7 ± 7.0	2.8 ± 1.2	7.7 ± 0.2	100.0 ± 94.9	5.7 ± 2.3	3.0 ± 1.0	1.5 ± 0.3	2.4 ± 1.5	65.9 ± 24.7	3.0 ± 2.3
Former smoker	11.9 ± 8.9	1.9 ± 0.6	4.5 ± 5.8	87.4 ± 82.9	4.2 ± 3.2	10.2 ± 5.4	2.7 ± 0.9	0.6 ± 0.3	70.9 ± 35.7	2.5 ± 0.9
Non smoker	11.4 ± 6.7	3.7 ± 2.9	7.2 ± 1.1	75.6 ± 72,7	4.3 ± 2.4	7.9 ± 5.8	1.8 ± 0.5	1.7 ± 1.1	38.9 ± 14.7	2.1 ± 1.0
p	0.752	0.595	0.749	0.486	0.148	0.006	0.030	0.054	0.103	0.710
Alcohol consumption										
Low	10.0 ± 6.4	2.9 ± 2.0	7.4 ± 1.0	60.5 ± 71.8	3.2 ± 1.9	5.8 ± 4.2	1.7 ± 0.2	2.1 ± 1.9		
Medium	9.5 ± 5.9	3.9 ± 3.0		83.2 ± 84.4	4.8 ± 2.3	5.4 ± 3.4	2.0 ± 0.7	1.7 ± 1.2	55.4 ± 34.8	2.3 ± 0.8
High	15.0 ± 7.3	2.5 ± 1.8	3.9 ± 5.0	84.0 ± 69.4	4.6 ± 2.8	9.9 ± 6.7	1.9 ± 0.8	1.5 ± 0.5	50.3 ± 25.1	2.4 ± 1.2
p	0.040	0.374	0.476	0.238	0.435	0.166	0.586	0.953	0.882	0.651
U-Cr end-shift (µg/g creatinine)										
1st tertile	10.3 ± 6.7	3.4 ± 2.8	6.6 ± 2.7	84.3 ± 83.6	4.7 ± 2.6	7.6 ± 5.7	1.9 ± 0.7	1.6 ± 1.2	51.0 ± 26.1	2.3 ± 1.1
2nd tertile	12.3 ± 6.7	3.1 ± 2.1		72.8 ± 62.9	4.1 ± 2.3	4.3 ± 1.8	1.6 ± 0.1	2.6 ± 1.7		
3rd tertile	16.8 ± 7.5			69.4 ± 58.4	3.2 ± 1.8					
p	0.219	0.391	0.245	0.798	0.578	0.436	0.670	0.325		

Table S3. Level of oxidative stress biomarkers parameters (mean ± SD) in groups of workers exposed to Cr(VI) and controls.

	Pre-shift			Post-shift		
	n	MDA (μg/L)	8-OHdG (μg/L)	n	MDA (μg/L)	8-OHdG (μg/L)
**Exposed Group**	**214**	**87.5 ± 78.0* £¥**	**4.77 ± 2.70£¥**	**213**	**76.5 ± 54.9**	**4.05 ± 2.64**
Bath plating workers	57	81.2 ± 45.2* £¥	4.92 ± 2.43£¥	57	72.7 ± 39.6	4.12 ± 2.63
Chromate paint applicators	8	82.4 ± 39.0£	4.12 ± 1.95£	8	70.6 ± 44.0	2.71 ± 0.92*
Welders	110	87.9 ± 96.0£¥	5.01 ± 3.10£¥	110	78.7 ± 55.0	4.17 ± 2.71
Machining workers	24	97.2 ± 64.1* £¥	4.09 ± 1.99£	23	88.8 ± 92.7	3.72 ± 2.05
Other activities	15	97.6 ± 74.6£	3.82 ± 1.55£	15	59.7 ± 30.5	4.11 ± 3.49
**Total control Group**	**84**	**71.3 ± 75.5**	**3.89 ± 2.53**	**-**	**-**	**-**
Within company control	69	75.8 ± 81.9	4.23 ± 2.62£	-	-	-
Outwith company control	15	51.0 ± 26.1	2.32 ± 1.09*	-	-	-
